# Ultra-low tidal volume ventilation during cardiopulmonary resuscitation shows no mitigating effect on pulmonary end-organ damage compared to standard ventilation: insights from a porcine model

**DOI:** 10.1186/s40635-023-00568-6

**Published:** 2023-11-25

**Authors:** Katja Mohnke, Philipp Conzelmann, Miriam Renz, Julian Riedel, René Rissel, Andrea Urmann, Johanna Hain, Bastian Duenges, Alexander Ziebart, Robert Ruemmler

**Affiliations:** Department of Anesthesiology, Medical Center of Johannes Gutenberg University, Langenbeckstrasse 1, 55131 Mainz, Germany

**Keywords:** Resuscitation, Ventilation, ULTVV, ARDS, MIGET

## Abstract

**Objective:**

This study aimed to determine whether ultra-low tidal volume ventilation (ULTVV) applied during cardiopulmonary resuscitation (CPR) compared with standard ventilation (intermittent positive pressure ventilation, IPPV) can reduce pulmonary end-organ damage in the post-resuscitation period.

**Methods:**

A prospective, randomized trial was conducted using a porcine model (*n* = 45). The animals were divided into three groups: IPPV, ULTVV, and a sham control group. Juvenile male pigs underwent CPR after inducing ventricular fibrillation and received the designated ventilation intervention [IPPV: tidal volume 6–8 ml per kilogram body weight (ml/kg BW), respiratory rate 10/min, FiO_2_ 1.0; ULTVV: tidal volume 2–3 ml/kg BW, respiratory rate 50/min, FiO_2_ 1.0]. A 20-h observation period followed if return of spontaneous circulation was achieved. Histopathological examination using the diffuse alveolar damage scoring system was performed on postmortem lung tissue samples. Arterial and venous blood gas analyses and ventilation/perfusion measurements via multiple inert gas elimination technique (MIGET) were repeatedly recorded during the experiment.

**Results:**

Out of the 45 experiments conducted, 28 animals were excluded based on predefined criteria. Histopathological analysis showed no significant differences in lung damage between the ULTVV and IPPV groups. ULTVV demonstrated adequate oxygenation and decarboxylation. MIGET measurements during and after resuscitation revealed no significant differences between the intervention groups.

**Conclusion:**

In the short-term follow-up phase, ULTVV demonstrated similar histopathological changes and functional pulmonary parameters compared to standard ventilation. Further research is needed to investigate the long-term effects and clinical implications of ULTVV in resuscitation settings.

**Supplementary Information:**

The online version contains supplementary material available at 10.1186/s40635-023-00568-6.

## Keypoints

The current state of research regarding the optimal ventilation strategy during cardiopulmonary resuscitation is incomplete. This study investigated the impact of ultra-low tidal volume ventilation during resuscitation compared to standard ventilation on lung end-organ damage. Histopathologically and functionally, no statistically significant differences were observed in the short-term follow-up.

**Supplementary Information:**

The online version contains supplementary material available at 10.1186/s40635-023-00568-6.

## Background

The optimal ventilation mode during ongoing cardiopulmonary resuscitation (CPR) remains undetermined among the resuscitation research community. There is a persisting lack of specific guideline recommendations regarding the ideal ventilation approach during cardiopulmonary resuscitation. Both the European Resuscitation Council (ERC) and the American Heart Association (AHA) do not offer detailed recommendations for ventilation during CPR [1, 2]. As a result, intermittent positive pressure ventilation (IPPV) has been habitually used for ventilation during ongoing resuscitation for decades, without studies ever demonstrating a significant survival advantage for this ventilation mode in particular.

Resuscitation guidelines recommend that an endotracheal tube may be used for airway protection if the user has sufficient airway expertise [1, 2]; this would allow for differentiated ventilation modes, such as ultra-low tidal volume ventilation (ULTVV) using tidal volumes of 2–3 ml per kilogram of bodyweight (ml/kg BW).

Ruemmler et al. [3] were able to demonstrate in a pilot study in a porcine model that this ventilation mode, which is already well-established in acute respiratory distress syndrome (ARDS) studies [4–6], showed advantages in terms of brain end-organ damage and did not exhibit any disadvantages regarding oxygenation during resuscitation and up to 6 h thereafter. Similarly, another animal study by this research group showed a reduced creatinine clearance during the short-term period after return of spontaneous circulation (ROSC) applying IPPV [7], suggesting potential end-organ damage mitigation by applying ULTVV. From a physiological point of view, the use of lower tidal volumes during mechanical ventilation is associated with a decrease in intrathoracic pressure [8]. This should improve venous return and blood flow [9] and lead to less lung inflammation in the context of lower alveolar distension [10].

However, there is still no answer to the question of how this ventilation mode impacts pulmonary end-organ damage during an extended post-ROSC observation phase.

Although cardiopulmonary resuscitation, by definition, is not considered a typical trigger of ARDS [11], ARDS occurs in nearly half (48%) of the patients who survive an out-of-hospital cardiac arrest beyond 48 h [12]. Since the occurrence of ARDS increases mortality and morbidity significantly during an intensive care stay after resuscitation [12], it is important to investigate whether the effects of lung-protective ventilation modes during CPR could prevent or at least ameliorate this.

Hence, the primary aim of this trial was to examine whether the use of ULTVV during CPR can mitigate histopathological damage of the lungs in comparison to standard ventilation (Intermittent Positive Pressure Ventilation) with tidal volumes of 8–10 ml/kg BW. As a secondary aim, it was investigated whether ULTVV during CPR has a beneficial effect on functional lung parameters, measured by Horovitz index, decarboxylation, and ventilation–perfusion distribution measured by multiple intert-gas elimination technique (MIGET).

## Materials and methods

### Anesthesia and instrumentation

This study was approved by the State and Institutional Animal Care Committee (Landesuntersuchungsamt Rheinland-Pfalz, Koblenz, Germany; approval no. G 16-1-042). The study is a prospective, randomized trial and was conducted from May 2019 to April 2020. The study followed the ARRIVE guidelines and involved 45 juvenile, male pigs (Sus scrofa domestica; mean weight 30 ± 3 kg; age 12–16 weeks) from a local breeder.

The experimental setup was based on previously conducted resuscitation studies [3, 13]. After intramuscular injection of ketamine (Hameln Pharmaceuticals GmbH, Hameln, Germany; 1.5 mg/kg BW), azaperone (Lilly Deutschland GmbH, Bad Homburg, Germany; 2.5 mg/kg BW), and midazolam (Hameln Pharmaceuticals GmbH, Hameln, Germany; 0.3 mg/kg BW), the sedated animals were transported to the laboratory.

An IV access was established at the ear. Anesthesia was then induced by a bolus injection of fentanyl (Janssen-Cilag, Neuss, Germany; 4 µg/kg BW) and propofol (Fresenius Kabi, Bad Homburg, Germany; 2 mg/kg BW), and a single dose of atracurium (HEXAL AG, Holzkirchen, Germany; 0.5 mg/kg BW) was administered to facilitate endotracheal intubation. The animals were ventilated in volume-controlled mode [tidal volume 6 ml/kg BW, positive end-expiratory pressure of 5 mbar, inspired fraction of oxygen (FiO_2_) of 0.4, inspiration to expiration ratio (I:E) of 1:2 and variable respiratory rate to achieve an end-tidal arterial pressure of carbon dioxide (PaCO_2_) below 6 kPa using an intensive care respirator (Engstroem care station, GE healthcare, Munich, Germany)]. Peripheral oxygen saturation was measured continuously with a sensor clipped to the ear (Radical 7, Masimo Corp., Irvine, CA, USA). General anesthesia was maintained using continuous infusions of fentanyl (0.1–0.2 mg/kg BW/h) and propofol (8–12 mg/kg BW/h). The animals received intravenous infusions of an isotonic electrolyte solution (Sterofundin^®^, B. Braun Melsungen AG, Melsungen, Germany) initially as a bolus of 30 ml/kg BW and then continuously with 5 ml/kg BW/h.

Arterial and central venous access was established using ultrasound guidance as previously described [13], and a pulse contour cardiac output system (PiCCO, Pulsion Medical Systems, Munich, Germany), a pulmonary artery catheter (PA Katheter Swan Ganz, Edwards Lifesciences Corporation, Irvine, CA, US), and a fibrillation catheter (VascoStim B 2/5F, Vascomed GmbH, Binzen, Germany) were inserted.

### Trial protocol and data collection

After the instrumentation, a half-hour consolidation phase followed, during which a set of six chemically inert gases with varying rates of transpulmonary elimination (sulfur hexafluoride, krypton, desflurane, enflurane, diethyl ether, and acetone) mixed in safe quantities with saline solution were injected intravenously. After allowing 30 min for stabilization and reaching a steady state, MIGET measurement (MIGET, MMIMS-System, Oscillogy LLC, Folsom, US) was conducted at the measurement time point baseline healthy (BLH). In this procedure, 10 ml of arterial and pulmonary arterial blood was drawn after heparinization of the blood, and the pulmonary elimination of inert gases was analyzed by mass spectrometry. Differences in transpulmonary elimination constants for those gases allow for a mathematical estimate toward ventilation/perfusion (V/Q) ratios and shunt measurement.

Arterial and mixed venous blood gases were obtained (radiometer, ABL90flex, Copenhagen, Denmark) at time point BLH. The hemodynamic parameters were recorded continuously using Datex Ohmeda S5 monitor (GE Healthcare, Munich, Germany) as well as the ventilation parameters using the internal software of the intensive care respirator.

The measurement time points after BLH are designated as follows: T4 denotes 4 h after ROSC, T20 denotes 20 h after ROSC, etc.

### Intervention

After BLH, the animals were randomized into one of three intervention groups by pulling sealed envelopes (Table 1). Ventricular fibrillation was induced via a venous fibrillation catheter, as previously described [13]. The sham group is the control group in which no cardiac arrest was induced, and accordingly, no ventilation intervention was performed.

**Table 1:** Group design and intervention parameters of the conducted experiments

Group	F_i_O_2_	Tidal volume [ml/kg BW]	Respiratory rate [^−min^]	I:E	PEEP [mbar]	*n*
IPPV	1.0	8–10	10	1:1	5	20
ULTVV	1.0	2–3	50	1:1	5	20
Sham	0.4	6–8	Variable^*^	1:2	5	5
n_total_						45

IPPV and ULTVV each describe the ventilation intervention during resuscitation. Sham refers to the control group, in which no cardiac arrest was induced and accordingly, no ventilation intervention either

*IPPV* intermittent positive pressure ventilation, *ULTVV* ultra-low tidal volume ventilation, *F*_*i*_*O*_*2*_ inspired fraction of oxygen, *I:E* inspiratory to expiratory ratio, *PEEP* positive end-expiratory partial pressure

^*^According to the desired target arterial partial pressure of carbon dioxide of < 6 Kilopascal

**Fig. 1:**
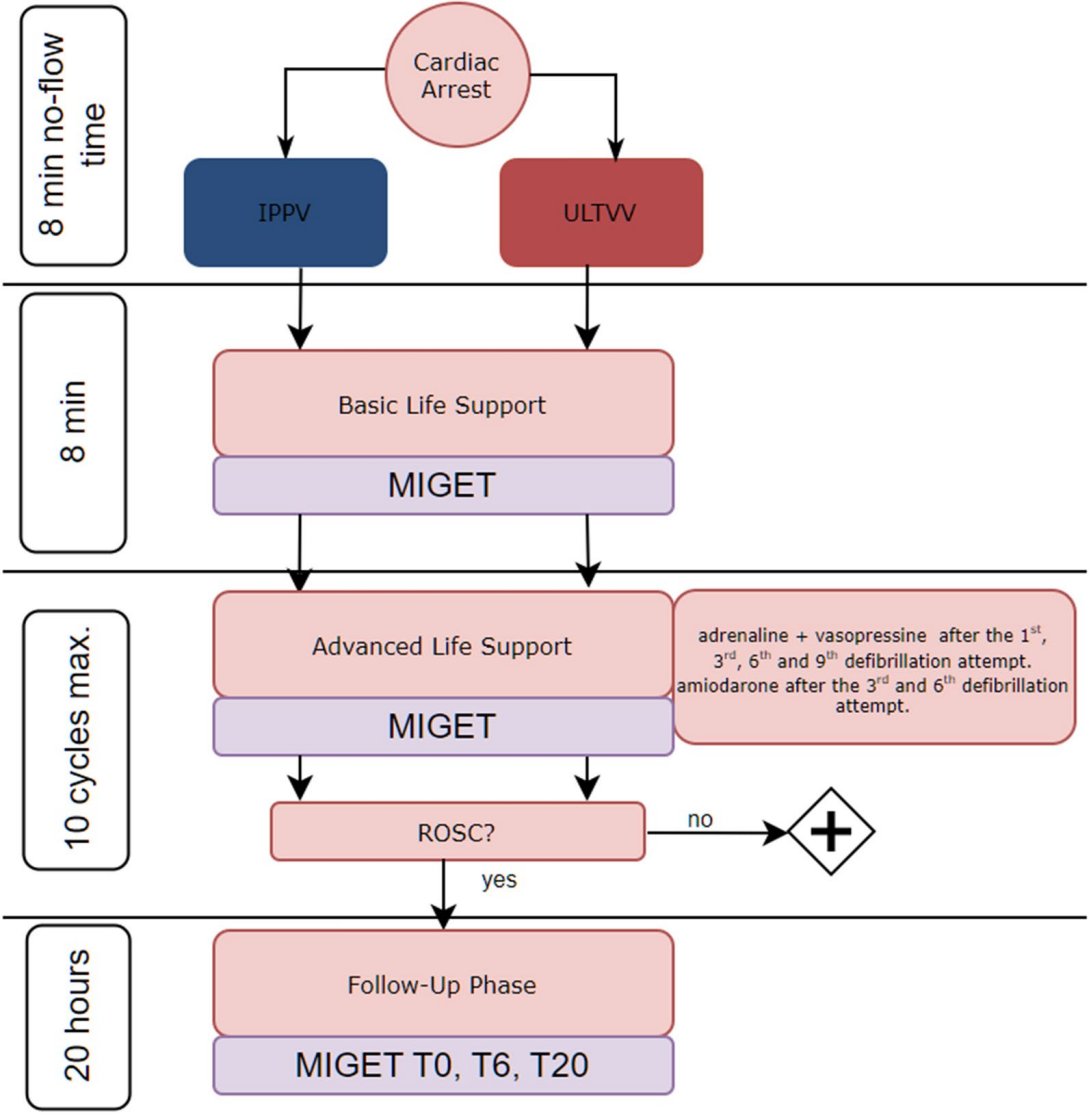
Flowchart of the intervention groups during the peri-resuscitation phase. If no ROSC was achieved, this led to exclusion from further data analysis. *ROSC* return of spontaneous circulation, *IPPV* intermittent positive pressure ventilation, *ULTVV* ultra-low tidal volume ventilation, *T*_*x*_ timepoint (in hours) after ROSC, *MIGET* multiple inert gas elimination technique. This figure was created using the graphics software draw.io

After monitor-confirmed ventricular fibrillation, ventilation was disconnected, and the animal was left untreated for 8 min. Eight minutes after induction of cardiac arrest, basic life support (BLS) was initiated: the animal was mechanically resuscitated with the assigned ventilation mode (Table 1). Thoracic compressions were administered using a mechanical chest compression device (LUCAS 2, Physio-Control, Redmond, US). Thoracic compressions were performed at a fixed frequency of 100 compressions per minute and a compression depth of 5 cm. After 8 min of BLS, an arterial and mixed venous blood gas analysis was taken, and blood samples were collected for MIGET. Immediately afterward, advanced life support (ALS) was performed, adapted to the ALS algorithm of the ERC [1]. If ventricular fibrillation or flutter was detected, immediate defibrillation was performed (200 Joule; Zoll R Series Monitor Defibrillator, Zoll Medical Deutschland GmbH, Cologne, Germany), resuscitation continued, and medications were administered intravenously according to the following schedule: adrenaline (1 mg; Sanofi-Aventis GmbH, Frankfurt a.M., Germany), vasopressin (0.5 U/kg BW; Pfizer Inc., New York City, US) after the first, third, sixth and ninth defibrillation, and amiodarone [150 mg; Hikma Pharma GmbH, Martinsried, (DE)] after the third and sixth defibrillation. After 8 min of ALS, arterial and mixed venous blood gas analysis, and MIGET measurement was performed (Fig. 1).

If return of spontaneous circulation (ROSC) was not achieved after ten ALS cycles, resuscitation was terminated, and the experiment was ended. If ROSC was achieved during a rhythm analysis, resuscitation was terminated, and the post-resuscitation phase began.

### Post-resuscitation phase

Animals which achieved ROSC were returned to standard ventilation, as described at baseline, and were monitored for 20 h with the aim of maintaining peripheral oxygen saturation above 93%. If necessary, the invasiveness of ventilation was adjusted according to the ARDS-network specifications [14], while mean arterial blood pressure was maintained above 60 mmHg using norepinephrine administration and volume boluses. Upon return of spontaneous circulation, the animals received a dose of 30 ml/kg BW of the electrolyte solution over a 2-h period, followed by an hourly rate of 2.5 ml/kg BW.

MIGET was taken again after 6 and after 20 h after ROSC. Blood gas analyses were performed repeatedly, and ventilation parameters were continuously recorded.

To prevent painful pressure sores, the animal was repositioned every 3–4 h between supine position, right and left side during the observation period.

The experiment was concluded by administering 40 mmol potassium chloride via the pulmonary artery catheter after inducing deeper general anesthesia with 200 mg propofol.

### Sample collection

Postmortem, the lungs were dissected free under preserved ventilation. Before the lungs were collected in their entirety, the trachea was clamped at the end of inspiration to minimize the risk of developing postmortem artificial atelectasis. Lung tissue samples were collected from the dorsal and ventral regions of the peripheral upper and lower lobes of the lung and fixed with 4% formalin. The samples were processed by the tissue bank at the University Medical Center Mainz in Germany, where they were paraffinized, sliced into 2-µm-thick sections, and then stained with hematoxylin–eosin (HE).

### Lung damage scoring system

Using an Olympus microscope (CX43RF, Olympus Cooperation, Tokyo, Japan) and CellSens software (Olympus cellSens Entry, Version 2.1, Olympus Corporation, Tokyo, Japan), the lung tissue samples were examined and assessed in a blinded fashion by a trained investigator using the established diffuse alveolar damage (DAD) scoring system [15], containing seven sub-items: alveolar edema, interstitial edema, hemorrhage, inflammation, epithelial damage, microatelectasis and overdistension. The DAD scoring system is the recommended examination tool of the expert consensus recommendations of the American Thoracic Society for simulating lung injury in animal models [16].

### Exclusion criteria

Only animals that showed no obvious signs of illness (e.g., normal eating and drinking behavior in the days before the experiment, no apparent injuries or inflammations) were included. This was ensured prior to sedation and transportation.

For animals in the intervention groups, only those who achieved sustained ROSC within the aforementioned study protocol were included in the data analysis.

### Statistical analysis

Statistical analyses were performed using one-way ANOVA with post-hoc Bonferroni correction [17], if the prerequisites for the use of ANOVA were given [18, 19]. Effects over time were analyzed using repeated measures ANOVA; within-subject effects were assessed using the Greenhouse–Geisser correction in case of non-sphericity. For single measurements, if normal distribution was not given, Kruskal–Wallis test [20] was used. Statistical evaluation was performed using IBM SPSS Statistics (IBM SPSS Statistics for Windows, Version 20. IBM Corporation, Armonk, NY, USA). The data are presented as mean (standard deviation). A significance level of 0.05 was set.

## Results

45 experiments were conducted. A total of 28 animals were excluded per protocol after no ROSC was achieved after ten ALS cycles. ROSC was achieved in six of the IPPV animals and six of the ULTVV animals, resulting in an overall ROSC rate of 30%. One of the IPPV animals died after achieving ROSC at time point T4 and was excluded from further data analysis. Five animals could be included as the sham comparison group.

Table 2 summarizes the respiratory and hemodynamic data and shows no differences between the groups at baseline.

**Table 2:** Hemodynamic and respiratory parameters in group comparison

Parameter	Group	Baseline	T6	T20
HR	IPPV	62 ± 11	85 ± 17	84 ± 50
	ULTVV	63 ± 15	93 ± 27	111 ± 59
	Sham	65 ± 9	73 ± 17	72 ± 21
MAP	IPPV	67 ± 8	64 ± 6*	75 ± 13
	ULTVV	73 ± 6	60 ± 6*	62 ± 13
	Sham	78 ± 5	79 ± 16	74 ± 25
CVP	IPPV	7 ± 2	9 ± 5	9 ± 3
	ULTVV	8 ± 2	7 ± 2	8 ± 3
	Sham	7 ± 1	6 ± 1	5 ± 1
NE^a^	IPPV	0 ± 0	0.069 ± 0.05*	0.044 ± 0.07*
	ULTVV	0 ± 0	0.187 ± 0.21*	0.971 ± 0.48*,#
	Sham	0 ± 0	0 ± 0	0 ± 0
CI^b^	IPPV	3.37 ± 0.56	2.81 ± 0.44	2.90 ± .71
	ULTVV	3.80 ± 1.13	3.63 ± 1.14	3.86 ± 1.10
	Sham	3.62 ± 0.73	3.25 ± 0.08	4.40 ± 1.61
PaO_2_	IPPV	212 ± 13	150 ± 46	173 ± 81
	ULTVV	198 ± 20	127 ± 17	93 ± 24
	Sham	189 ± 15	143 ± 32	103 ± 23

Mean (± SD). Group effects are analyzed by univariate ANOVA with post-hoc Bonferroni correction and in the case of norepinephrine by Kruskal–Wallis. There were no intergroup differences at baseline. n(IPPV) = 5; n(ULTVV) = 6; n(sham) = 5

*HR* heart rate, *MAP* mean arterial pressure, *CVP* central venous pressure, *NE* norepinephrine dosage, *CI* cardiac index, *PaO*_*2*_ arterial partial pressure of oxygen, *T*_*x*_ time point, *IPPV* intermittent positive pressure ventilation, *ULTVV* ultra-low tidal volume ventilation

^a^[µg kg BW^−1^ min^−1^]

^b^(l min^−1^) m^2−1^

^*^p < 0.05 intervention group vs. sham at the given time point

^#^*p* < 0.05 IPPV vs. ULTVV at the given time point

### Histopathological damage

**Fig. 2:**
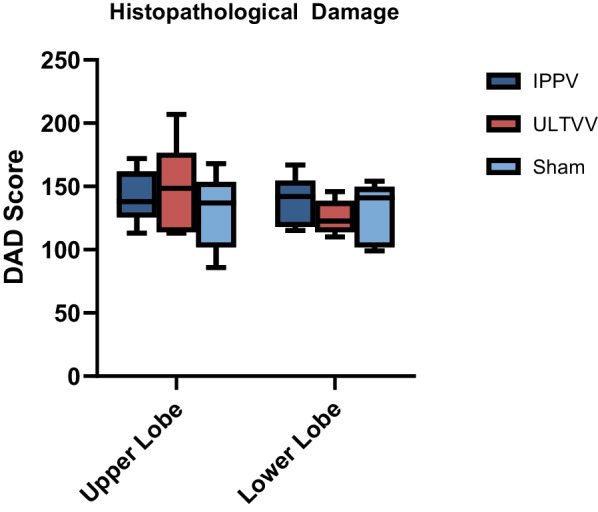
Histopathological evaluation of the lung. Diffuse alveolar damage score—separate evaluation by upper and lower lobes. No statistically significant differences between the groups. *DAD* diffuse alveolar damage, *IPPV* intermittent positive pressure ventilation, *ULTVV* ultra-low tidal volume ventilation

The analysis of postmortem lung tissue samples showed no significant differences between the groups (*p* > 0.562). Generally, the upper lobe of the intervention groups tends to show more signs of tissue damage than the control group, but without a statistically significant difference (Fig. 2). There were also no statistically significant differences between the groups regarding the individual items of the lung injury scoring system (*p* > 0.116) (Table 3).

**Table 3:** Individual items of the DAD score—overall assessment of the lung

Parameter	Group	Mean (± SD)	*p*
Alveolar edema	IPPV	19.2 (± 2.4)	0.942
	ULTVV	17.5 (± 10.4)	
	Sham	18.2 (± 8.6)	
Interstitial edema	IPPV	55.6 (± 9.5)	0.897
	ULTVV	53.8 (± 6.2)	
	Sham	53.0 (± 11.1)	
Hemorrhage	IPPV	17.6 (± 5.1)	0.530
	ULTVV	17.7 (± 14.1)	
	Sham	11.4 (± 7.0)	
Inflammation	IPPV	46.8 (± 5.6)	0.269
	ULTVV	51.2 (± 3.4)	
	Sham	55.0 (± 10.8)	
Epithelial damage	IPPV	64.6 (± 7.0)	0.217
	ULTVV	59.2 (± 9.0)	
	Sham	52.4 (± 14.2)	
Microatelectasis	IPPV	30.2 (± 7.0)	0.503
	ULTVV	31.0 (± 5.8)	
	Sham	35.6 (± 10.2)	
Overdistension	IPPV	45.8 (± 9.5)	0.116
	ULTVV	44.5 (± 7.5)	
	Sham	32.8 (± 13.1)	

Group effects are analyzed by univariate ANOVA with post-hoc Bonferroni correction. There was no statistically significant difference between the groups in the individual items of the DAD score. n(IPPV) = 5; n(ULTVV) = 6; n(sham) = 5

*DAD* diffuse alveolar damage, *IPPV* intermittent positive pressure ventilation, *ULTVV*: ultra-low tidal volume ventilation

### Oxygenation and decarboxylation

**Fig. 3:**
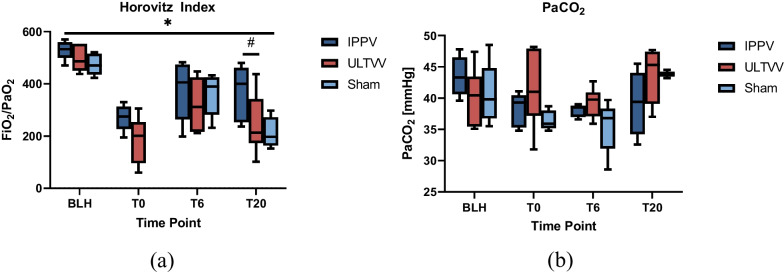
Oxygenation and ventilation parameters. **a** Horovitz index as a function of time. The Horovitz index significantly decreases during the course of the experiment in all groups without group differences. **b** Partial pressure of carbon dioxide as a function of time. There is no significant difference between the groups. *p < 0.05 (Horovitz index BLH vs. following timepoints for all groups). #*p* < 0.05 (Horovitz index IPPV vs. ULTVV at T20). *BLH* baseline healthy, *IPPV* intermittent positive pressure ventilation, *ULTVV* ultra-low tidal volume ventilation, *T*_*x*_ timepoint, *paCO*_*2*_ partial pressure of carbon dioxide

The Horovitz index (PaO_2_/FiO_2_) decreased significantly over time in all groups (*p* < 0.001) without group difference (*p* = 0.059) (Fig. 3a). Neither during basic life support (*p* = 0.667) nor during advanced life support (*p* = 0.157) were there differences in Horovitz index. At time T20, there was a significant group difference for IPPV vs ULTVV (*p* = 0.018).

The PaCO_2_ values showed no significant differences during resuscitation (*p* > 0.480). The PaCO_2_ values showed no difference after ROSC (*p* = 0.260) (Fig. 3b).

### MIGET

**Fig. 4:**
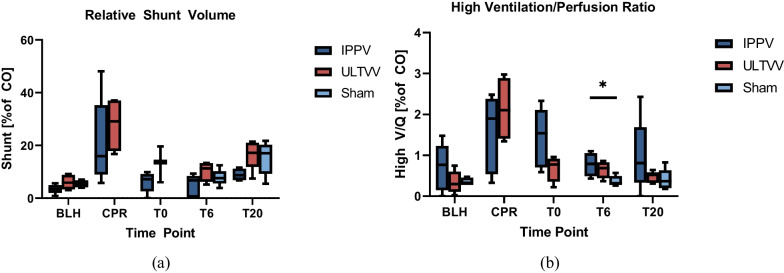
MIGET measurements. **a** Relative shunt volumes, expressed as a percentage of cardiac output (CO), serve as an indicator for a ventilation/perfusion mismatch and the diversion of pulmonary gas exchange through circulatory bypass. **b** The ratio of high V/Q units, represented as a percentage of cardiac output (CO), indicates lung areas that are over-ventilated but have inadequate blood flow to effectively participate in gas exchange. *p < 0.05 (IPPV vs. sham at T6). *BLH* baseline healthy, *CPR* cardiopulmonary resuscitation, *IPPV* intermittent positive pressure ventilation, *ULTVV* ultra-low tidal volume ventilation, *CO* cardiac output, *T*_*x*_ timepoint, *MIGET* multiple inert gas elimination technique

Ventilation/perfusion analyses showed neither any difference in pulmonary shunt during (p = 0.327) and immediately post-ROSC (*p* = 0.86) (Fig. 4a) nor any difference in hyperventilated lung areas (high V/Q) during CPR (*p* = 0.394) (Fig. 4b). At time T6, there was a statistically significant difference for high V/Q for IPPV vs. sham (*p* = 0.032). The individual measurements of the MIGET can be found in Additional file 1: Table S1.

### Accuracy of the ventilation intervention

During BLS, peak inspiratory pressure and mean airway pressure were significantly higher for the IPPV group (*p* = 0.027 and *p* = 0.022) (Additional file 1: Fig. S1a and b). Driving pressure was significantly higher for IPPV during basic life support (*p* = 0.021) (Additional file 1: Fig. S1c).

## Discussion

In the present study, the influence of ultra-low tidal volume ventilation compared to standard ventilation during cardiopulmonary resuscitation and with a control group was investigated with an observation period of 20 h after return of spontaneous circulation, focusing on histopathological lung damage and the clinical impairment of the pulmonary function.

After 20 h of follow-up, there were no statistically significant histopathological differences between the intervention groups, nor with the control group. Similarly, decarboxylation and ventilation–perfusion-measurements using the multiple inert gas elimination technique did not differ during the course of the trial.

An established porcine CPR model [13, 21, 22] was applied for the first time, to the best of our knowledge, to investigate the influence of the application of low-tidal volume ventilation during CPR under controlled conditions in a prolonged post-resuscitation period. The effects of this ventilation intervention were examined using validated measures such as the DAD scoring system [16, 23] and MIGET technology. MIGET is a technique that allows us to look more closely at lung function in animal models, specifically the ventilation/perfusion (V/Q) ratio and the shunt fraction of cardiac output. Previously published trials [3, 24, 25] have validated the application of MIGET technology during CPR, which can offer supplementary insights into circulation and ventilation impairment during CPR.

In contrast to the pilot study [3], no significant group differences were observed in terms of the histological assessment of lung tissue damage using the DAD score.

For the individual criteria, there is a tendency toward increased overinflation (IPPV > ULTVV > sham), epithelial damage (IPPV > ULTVV > sham), and hemorrhage (IPPV = ULTVV, both > sham) in the resuscitated animals compared to the sham group, although these differences did not reach statistical significance. These tendencies can be explained by the mechanical stress of cardiopulmonary resuscitation and the potential hemorrhage, which may also be influenced by the administration of heparin.

Potentially, the compensatory increase in respiratory rate in this model, which is intended to ensure adequate decarboxylation, despite the lower tidal volumes in ULTVV might result in a similarly high mechanical power [26], leading to comparable stress on lung tissue as in IPPV. Future studies should investigate the influence of different high respiratory rates at reduced tidal volumes on mechanical power and lung injury, possibly at the expense of reduced decarboxylation capacity of the whole organism.

In the present model, the airway was secured using an endotracheal tube before the onset of cardiovascular arrest and consecutively before the start of chest compressions. This makes aspiration of gastric contents very unlikely. However, regurgitation of acidic gastric contents after the onset of unconsciousness and consecutive aspiration are a presumed cause of ARDS after CPR in clinical reality [12, 27, 28]. This is a possible explanation for the generally not very pronounced lung damage found in this study.

In this study, the significantly increased Horovitz index observed in the ULTVV group compared to the IPPV group in the previous study [3] could not be reproduced during the post-resuscitation phase. During resuscitation, there was no statistically significant difference between ULTVV and IPPV in terms of Horovitz index. This is noteworthy because theoretically, the low tidal volume and compensatory increased respiratory rate could result in higher dead space ventilation, which in turn could have a negative impact on gas exchange [29]. The Horovitz index decreased significantly toward the end of the experiment in all groups. The Horovitz index was significantly higher for IPPV than for ULTVV at the end of the observation period. This was attributed to the fact that in two animals in the IPPV group, ventilatory invasiveness, namely PEEP and FiO_2_, had to be increased relevantly toward the end of the experiment because they fell below the predefined threshold for peripherally measured oxygen saturation of 93%. As a result, as described in the study protocol, the invasiveness of ventilation was increased according to the ARDS network table [14]. This resulted in improved PaO_2_ and consequently an improved Horovitz index, under the expense of increased ventilatory invasiveness. After pulmonary function deteriorated relevantly during the course of the experiment, particularly in the IPPV group—to the extent that ventilatory intervention became necessary—oxygenation nominally increased after appropriate intervention. Thus, it appears that the oxygenation performance of the IPPV group was best—tended to do even better than the sham group, which was not exposed to the stress of cardiopulmonary resuscitation. We contemplated that this was primarily due to the necessary increase in PEEP [30]. The increase in PEEP may also have resulted in decreased V/Q mismatches [31], which is discussed in more detail below. The fact that the ventilatory invasiveness was not constant in the groups means that a clear effect of the ventilatory intervention cannot be concluded here.

In contrast to the previous studies [3, 24], the current investigations did not reveal significant differences in terms of shunt volume and high V/Q ratios based on the MIGET measurements among the intervention groups. It is noteworthy that both intervention groups had a minimal proportion of hyperventilated lung areas (high V/Q ratio) of 3.0% or less. These values are comparable to those of Ruemmler et al. [3] in which, in the porcine resuscitation model, the IPPV group had 3.5% and the ULTVV group 1.1% high V/Q-ratio. In contrast, in Hartmann et al. [24], the IPPV group showed a high V/Q ratio of 42% in the porcine resuscitation model, despite a similar experimental setup. Furthermore, this proportion decreases from the CPR to ALS time point. We contemplated that this difference could be explained by the use of vasopressin, which was not utilized by Hartmann et al. [24] and generally improves perfusion [32]. Immediately after ROSC (T0), there is a tendency for increased hyperinflation in the IPPV group compared with the UTLVV group; at time T6, hyperinflation is statistically significantly increased for IPPV compared with sham. This could be an indication of worsened gas exchange and blood flow in the post-resuscitation phase for IPPV. Shunt and high V/Q ratio showed no statistically significant difference between groups at the end of the follow-up period.

In the present study, the rates of ROSC achieved in previous studies were not reached [3, 24]. We attribute the decreased rates of ROSC to the significantly longer no-flow time employed in this study [22].

The significant group differences in peak airway pressure, driving pressure, and mean airway pressure during resuscitation can be attributed to the differences in ventilation modes and are thus to be expected [3], indicating that the ventilation intervention was performed correctly.

Unlike in the study by Ruemmler et al. [3], these differences did not translate into statistically significant differences between the intervention groups in the other parameters investigated during resuscitation itself, the post-resuscitation phase, or the postmortem lung histology.

It is notable that at time T20, significantly higher norepinephrine drip rates were required to maintain a mean arterial blood pressure above 60 mmHg for the ULTVV group than for the IPPV group. Circulatory instability presented inhomogeneously in the post-resuscitation period. Given the relatively low rate of ROSC and that there is a multitude of reasons for cardiovascular instability post-resuscitation [33–35], we do not assume that this is an effect of the ventilation intervention.

### Limitations

Our study has several limitations:

The resuscitation rates from previous studies and the pilot study (ROSC rate: 66%) [3, 24] were used for empirical sample size estimation. However, these rates were not achieved, and the small group size limits the validity of the findings substantially. Adequately powered large animal studies pose a general challenge, since conducting larger numbers of experiments requires excessive resources, often leading to underpowered empirical study designs. This is a known problem and additionally weakens the conclusions that can be derived from most large animal trials.

In the pilot study [3], only a no-flow time of 4 min was observed before commencing basic life support. In the present study, a no-flow time of 8 min was initiated. This represents a more realistic simulation of out-of-hospital cardiac arrest [36], but it results in lower rates of ROSC [22]. Nevertheless, these 8 min may not have been sufficient to adequately induce ischemia–reperfusion injury, resulting in the statistically non-significant differences in histopathologic lung injury. However, longer no-flow times would have resulted in even worse ROSC rates and, when applied translationally, may be associated with a disastrous neurological outcome [37].

Some medications were administered that are not typically found in a human medical setting. Vasopressin was used to optimize the rates of ROSC [32]. This has the following effects: vasopressin improves end-organ perfusion and may as a result influence V/Q measurements [24]. However, this has not been observed as being clinically relevant in previous trials of our research group.

For the MIGET measurement, 3000 IU of Heparin was administered systemically to prevent the formation of blood clots during blood sampling at the MIGET measurement time points. This could potentially increase the risk of bleeding and affect histological evaluation. However, since the experimental animals exposed to the massive mechanical stress of cardiopulmonary resuscitation [38] did not exhibit significantly elevated values in the sub-item hemorrhage of lung damage concerning the DAD scoring system or showed any macroscopic hemorrhage during necropsy, the effect appears to be negligible in this study.

In addition, automated chest compression devices are used in only 10–14% of cases in human medicine [39], as their routine use is not justified by a general survival advantage [40]. However, in the presented experiment, their use serves to standardize external chest compressions and to improve the comparability of ventilation interventions.

The MIGET measurement has proven itself in the past as a useful tool for conducting V/Q measurements during resuscitation [24]. Even so, it is a highly sensitive procedure. Repeating a measurement after a preceding erroneous measurement is not possible due to the tightly scheduled experimental setup. As a result, even minor changes in gas solubility can lead to significant deviations in the results. Therefore, the result of an MIGET measurement always represents an approximation of the actual V/Q ratio. 

By definition, ARDS manifests within 1 week after an injurious event. Due to local regulations, the experimental procedure is limited to 24 h. As a result, the post-observation period in the current experiment may have been insufficient to observe the full extent of pulmonary damage and potential group differences.

### Clinical significance

This study did not find a significant difference in histopathological or functional lung damage related to the ventilation intervention. However, due to the expected reduced invasiveness of ventilation due to decreased tidal volumes during ongoing resuscitation, it is plausible to consider that ULTVV could have potential mitigating effects on pulmonary damage and the incidence of ARDS, which might ultimately improve morbidity and mortality rates. Nonetheless, within the confines of this specific experimental setup, we did not observe a definitive beneficial effect. To establish such effects in a clinical context, further investigation involving human subjects would be necessary.

### Supplementary Information

**Additional file 1: Figure S1.** Ventilation pressures as a function of time at BLH and during CPR. **a** Peak inspiratory pressure. **b** Mean airway pressure. **c** Driving pressure. During BLS, the ventilation pressures for the IPPV group are significantly higher than for the ULTVV group. *indicates *p* < 0.05 (IPPV vs. ULTVV at time point BLS). *IPPV* intermittent positive pressure ventilation, *ULTVV* ultra-low tidal volume ventilation, *BLH* baseline healthy, *BLS* basic life support, *ALS* advanced life support. **Table S1.** Individual measurements of MIGET.

## Data Availability

All relevant data obtained and analyzed during this study are presented in the main text and the supplement.

## Abbreviations

AHA: American Heart Association
ALS: Advanced life support
ARDS: Acute respiratory distress syndrome
BLH: Baseline healthy
BLS: Basic life support
CO: Cardiac output
CPR: Cardiopulmonary resuscitation
CVP: Central venous pressure
DAD: Diffuse alveolar damage
ERC: European Resuscitation Council
FiO_2_: Fraction of inspired oxygen
h: Hour
HE: Hematoxylin–eosin
I:E: Inspiration to expiration ratio
IPPV: Intermittent positive pressure ventilation
kPa: Kilopascal
MAP: Mean arterial pressure
mbar: Millibar
MIGET: Multiple inert gas elimination technique
mg/kg BW: Milligrams per kilogram bodyweight
ml/kg BW: Milliliters per kilogram bodyweight
µg/kg BW: Micrograms per kilogram bodyweight
mmHg: Millimeters of mercury
NE: Norepinephrine
PA: Pulmonary artery
PaCO_2_: Partial pressure of carbon dioxide
PaO_2_: Partial pressure of oxygen
PEEP: Positive end-expiratory pressure
PiCCO: Pulse index continuous cardiac output
ROSC: Return of spontaneous circulation
T_x_: Time point
U: Units
ULTVV: Ultra-low tidal volume ventilation
V/Q: Ventilation/perfusion ratio
