# Supplementary material for: Ultra-low tidal volume ventilation during cardiopulmonary resuscitation shows no mitigating effect on pulmonary end-organ damage compared to standard ventilation: insights from a porcine model
Source: Intensive Care Med Exp. 2023 Nov 25;11:81. doi: 10.1186/s40635-023-00568-6 (PMC10676323; doi:10.1186/s40635-023-00568-6)
Supplement: Supplementary file 1 — Additional file 1: Figure S1. Ventilation pressures as a function of time at BLH and during CPR. a Peak inspiratory pressure. b Mean airway pressure. c Driving pressure. During BLS, the ventilation pressures for the IPPV group are significantly higher than for the ULTVV group. *indicates p < 0.05 (IPPV vs. ULTVV at time point BLS). IPPV intermittent positive pressure ventilation, ULTVV ultra-low tidal volume ventilation, BLH baseline healthy, BLS basic life support, ALS advanced life support. Table S1. Individual measurements of MIGET. [file 40635_2023_568_MOESM1_ESM.docx]

Additional file

|  |  |
| --- | --- |
| (a) | (b) |
|  |  |
| (c) |  |

**Figure S1.** Ventilation pressures as a function of time at BLH and during CPR. (a) Peak inspiratory pressure. (b) Mean airway pressure (c) Driving pressure. During BLS, the ventilation pressures for the IPPV group are significantly higher than for the ULTVV group. * indicates p < 0.05 (IPPV vs. ULTVV at timepoint BLS). *IPPV* intermittent positive pressure ventilation, *ULTVV* ultra-low tidal volume ventilation, *BLH* baseline healthy, *BLS* basic life support, *ALS* advanced life support.

**Table S1**. Individual measurements of MIGET

| **measuring method** | **subject** | **BLH** | **CPR** | **T0** | **T6** | **T20** |
| --- | --- | --- | --- | --- | --- | --- |
| shunt | IPPV1 | 4.19 | 22.33 | 8.48 | 7.4 | 7.39 |
| (% of CO) | IPPV2 | 3.31 | 12.23 | 9.8 | 9.33 | 11.58 |
|  | IPPV3 | 5.62 | 48.11 | 5.2 | 0.57 | 10.04 |
|  | IPPV4 | 0.88 | 5.78 | 0.0 | 0 | 75.23* |
|  | IPPV5 | 3.44 | 15.93 | 7.18 | 6.79 | 6.7 |
|  | ULTVV1 | 3.05 | 16.72 | 6.03 | 5.12 | 7.42 |
|  | ULTVV2 | 7.51 | 36.81 | 13.68 | 9.61 | 17.22 |
|  | ULTVV3 | n.m. | n.m. | n.m. | n.m. | n.m. |
|  | ULTVV4 | 9.17 | 37.08 | 53.38* | 13.38 | 21.42 |
|  | ULTVV5 | 4.25 | 21.47 | 19.62 | 13.04 | 20.49 |
|  | ULTVV6 | 71.1* | n.m. | n.m. | n.m. | 16.46 |
|  | Sham1 | 3.9 | n.m. | n.m. | 3.87 | 5.46 |
|  | Sham2 | 5.08 | n.m. | n.m. | 7.29 | 17.02 |
|  | Sham3 | 7.02 | n.m. | n.m. | 7.59 | 21.75 |
|  | Sham4 | 6.12 | n.m. | n.m. | 7.85 | 18.69 |
|  | Sham5 | n.m. | n.m. | n.m. | 12.48 | 13.12 |
| high V/Q | IPPV1 | 0.3 | 2.28 | 0.59 | 0.67 | 0.67 |
| (% of CO) | IPPV2 | 0.77 | 2.48 | 1.54 | 0.91 | 0.81 |
|  | IPPV3 | 0.98 | 0.75 | 2.33 | 0.43 | 2.43 |
|  | IPPV4 | 0 | 0.33 | 1.89 | 57.27* | 0 |
|  | IPPV5 | 1.48 | 1.9 | 0.83 | 1.1 | 0.94 |
|  | ULTVV1 | 0.75 | 2.98 | 0.78 | 0.86 | 0.64 |
|  | ULTVV2 | 0.45 | 1.61 | 0.96 | 0.65 | 0.35 |
|  | ULTVV3 | n.m. | n.m. | n.m. | n.m. | n.m. |
|  | ULTVV4 | 0.3 | 1.34 | 0.22 | 0.73 | 0.52 |
|  | ULTVV5 | 0.19 | 2.6 | 0.77 | 0.37 | 0.31 |
|  | ULTVV6 | 0.03 | n.m. | n.m. | n.m. | 0.53 |
|  | Sham1 | 0.48 | n.m. | n.m. | 0.57 | 0.83 |
|  | Sham2 | 0.37 | n.m. | n.m. | 0.3 | 0.22 |
|  | Sham3 | 0.28 | n.m. | n.m. | 0.41 | 0.37 |
|  | Sham4 | 0.29 | n.m. | n.m. | 0.32 | 0.18 |
|  | Sham5 | n.m. | n.m. | n.m. | 0.26 | 0.44 |

n.m. means that there is no measurement for the corresponding subject at this time. This is either because, according to the study protocol, no measurement was required at this time (e.g. for sham at the time points CPR and T0) or because no measurement could be performed (e.g. due to a device defect). * is an outlier that we most likely attribute to measurement error. We have excluded this from the data analysis. *MIGET* multiple inert gas elimination technique. *ULTVV* ultra-low tidal volume ventilation, *CO* cardiac output, *BLH* baseline healthy, *CPR* cardiopulmonary resuscitation *T_x_*: timepoint, *MIGET* multiple inert gas elimination technique. *high V/Q* hyperventilated lung areas, *n.m.* no measurement.
